# Llamas use social information from conspecifics and humans to solve a spatial detour task

**DOI:** 10.1007/s10071-023-01808-8

**Published:** 2023-07-06

**Authors:** Annkatrin Pahl, Uta König von Borstel, Désirée Brucks

**Affiliations:** 1 Department of Anthropology/Sociobiology, University of Göttingen, Göttingen, Germany; 2 Institute of Behavioural Physiology, Research Institute for Farm Animal Biology (FBN), Dummerstorf, Germany; 3 Animal Husbandry, Behaviour and Welfare Unit, Department of Animal Breeding and Genetics, University of Giessen, Giessen, Germany

**Keywords:** Domestication, Emulation, Human demonstration, New world camelids, Social learning, Stimulus enhancement

## Abstract

Learning by observing others (i.e. social learning) is an important mechanism to reduce the costs of individual learning. Social learning can occur between conspecifics but also heterospecifics. Domestication processes might have changed the animals’ sensitivity to human social cues and recent research indicates that domesticated species are particularly good in learning socially from humans. Llamas (*Lama glama*) are an interesting model species for that purpose. Llamas were bred as pack animals, which requires close contact and cooperative behaviour towards humans. We investigated whether llamas learn socially from trained conspecifics and humans in a spatial detour task. Subjects were required to detour metal hurdles arranged in a V-shape to reach a food reward. Llamas were more successful in solving the task after both a human and a conspecific demonstrated the task compared to a control condition with no demonstrator. Individual differences in behaviour (i.e. food motivation and distraction) further affected the success rate. Animals did not necessarily use the same route as the demonstrators, thus, indicating that they adopted a more general detour behaviour. These results suggest that llamas can extract information from conspecific and heterospecific demonstrations; hence, broadening our knowledge of domesticated species that are sensitive to human social behaviour.

**Supplementary Information:**

The online version contains supplementary material available at 10.1007/s10071-023-01808-8.

## Introduction

To acquire novel behaviours, animals often rely on trial-and-error learning; however, particularly in unpredictable and rapidly changing environments or other costly situations (Smolla et al. 2015), learning behaviours from experienced conspecifics might be beneficial (Heyes 1994). Social learning describes the acquisition of information by observing or interacting with others (Galef & Laland 2005) and can be found across animal taxa ranging from primates (Whiten and van de Waal 2017) to insects (Leadbeater and Chittka 2007). Information is usually acquired from older or experienced individuals within the social group but sometimes heterospecifics can serve as source of information as well. Learning by observing individuals of another species can be beneficial, for example, when interspecific competition is high (Seppänen et al. 2007). Usually heterospecific social learning is observed between species that share the same habitat, feeding ecology or predation pressures (Avarguès-Weber et al. 2013). Due to increasing human presence worldwide, humans are a part of many, if not most, species’ natural environments to date. Humans are treated either as irrelevant or threatening for other species (Goumas et al. 2020); however, some species see humans as a positive aspect of their environment (Rault et al. 2020). Domesticated species not only share their environment with humans but also depend on humans for survival (e.g. food, shelter, breeding, etc.). Accordingly, it would be advantageous for domesticated animals to pay close attention to human behaviour as a source of information about the environment that allows to predict events, such as feeding times or locations (see Hopper 2021 for a review). Recent studies have shown that domestic species (geese: Fritz et al. 2000; goats: Nawroth et al. 2016a; dogs: e.g. Pongrácz et al. 2001; horses: e.g. Schuetz et al. 2017, but see Burla et al. 2018) seem to be able to learn socially from human demonstrations, while non-domesticated species (e.g. dingoes: Smith and Litchfield 2010) were not, except for when they experienced intense socialisation with humans (e.g. wolves: Range and Virányi (2013); chimpanzees: Ross et al. (2010), Hopper et al. (2015); dolphins: Kuczaj and Yeater (2007)). Consequently, it has been hypothesised that domestication affected the cognitive abilities necessary for communicating with humans (Hare and Tomasello 2005; Udell et al. 2010; see Jardat and Lansade 2021 for a review), such as understanding of human gestures (e.g. Hare et al. 2002; but see Range and Virányi 2011; Udell et al. 2008), emotions (e.g. Smith et al. 2016), attentional states (e.g. Proops and McComb 2010), and gaze (e.g. Nawroth et al. 2016b).

Research on the effects of domestication on cognitive abilities so far had a major focus on companion animals (i.e. dogs (Lea and Osthaus 2018)); however, many other species have been domesticated for various purposes, and testing these may ultimately broaden our knowledge about the effects of domestication on behaviour (Jardat and Lansade 2021). One taxon that has received very little attention are New World camelids (Lamini) to which the genera Lama belongs to (Miranda-de la Lama and Villarroel 2023). Llamas (*Lama glama*) were domesticated from Guanacos (*Lama guanicoe*) around 3800–5000 years ago (Yacobaccio and Vilá 2016), and were primarily used as pack animals while also providing meat and to a lesser extend fibre (Mengoni Goñalons and Yacobaccio 2006). Llamas used to accompany humans in the Andes on trade caravans (Browman 1974) and, thus, were potentially selected for a cooperative and less reactive temperament. Llamas are adapted to a wide range of environmental conditions, such as deserts, mountain plateaus and steppe (Wheeler 1995). In order to survive in these different ecological conditions with varying locations, quality and quantity of resources, llamas exhibit high degrees of foraging flexibility (Pfister et al. 1989) and need to rely on one another for communal defence against predators (Taraborelli et al. 2012). Consequently, it might be beneficial to socially transmit information about predators but potentially also about high-quality foraging sites between group members. Additionally, considering the close association and frequent interactions with humans, it might have been beneficial for llamas to pay close attention to humans as well. Indeed, previous studies have shown that llamas can discriminate between humans (Taylor and Davis 1996) and follow the gaze of conspecifics and humans into distant space, while guanacos failed to exhibit gaze-following in the same study (Schaffer et al. 2020). Furthermore, they are able to form social relationships with humans through early positive interactions and humans can act as a social buffer during potentially stressful situations such as shearing (Windschnurer et al. 2020).

In the current study, we assessed llamas’ hetero- and conspecific social learning abilities in a spatial detour task. Llamas were assigned to observe either a human demonstrator, a conspecific demonstrator or no demonstrator (control) solving a detour task before they could solve the task themselves. We hypothesised that llamas are able to learn socially from humans and conspecifics, and accordingly, individuals in the two demonstration groups were expected to be more successful compared to the control group.

## Methods

### Subjects and housing

The study was carried out on three different farms in Germany. In total, we tested 43 adult llamas (19 females and 24 males) with an age of 2–18 years (see Supplementary Data). All animals were kept for regular touristic trekking activities and therefore were used to being led on a halter by unfamiliar people. The animals were housed in social groups (either same-species or mixed-species groups with alpacas) and were not food restricted before testing.

### Procedure

**Fig. 1:**
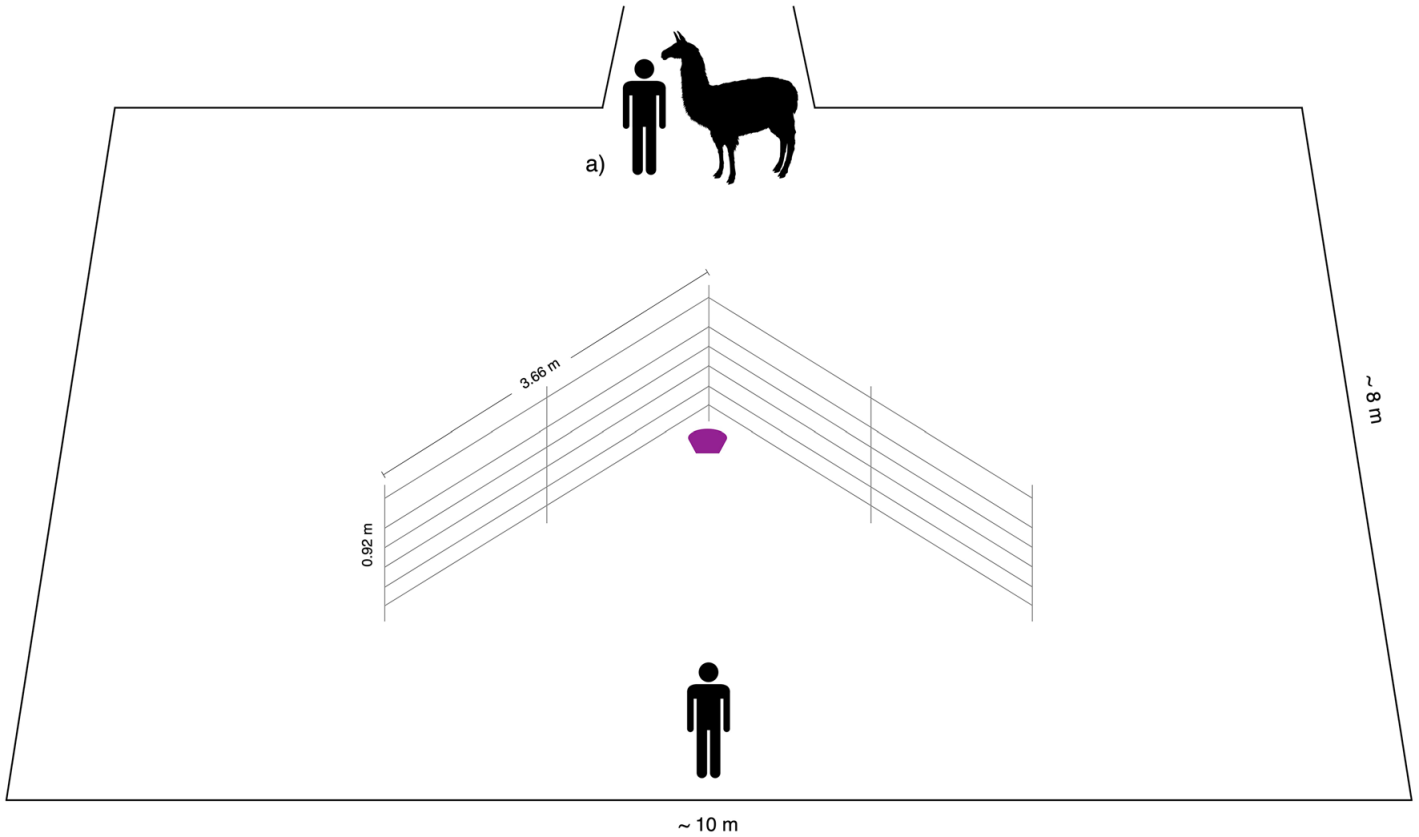
Experimental arena (approx. 8 × 10 m) with V-shaped metal hurdles. The animal is depicted at the starting point (**a**). The food reward is placed in the purple bowl behind the hurdle.

The experiments were carried out in an arena (approx. 8 × 10 m) that was built up adjacent to the home pens of the animals to spatially isolate the test individual. To reduce potential distress, we ensured that visual, auditory and olfactory contact with familiar animals was possible throughout the test. To avoid differences in learning opportunities, only animals which either already finished the test or were not included in the study, had visual access to the arena during testing. For all three farms, these bystanders were positioned in the back of the hurdles behind the experimenter. Two metal hurdles (length: 366 cm, height: 92 cm) were arranged in a V-shaped manner and placed in the middle of the test arena (Fig. 1). All tests were conducted by two female experimenters, both unfamiliar to the animals. One experimenter handled the animals during the test (“handler”, AP), while the other experimenter placed the food bowl on the ground and acted as the human demonstrator (“experimenter”, DB). Each subject was led to the starting position and was held on a leash attached to the halter until the test started. All tests were recorded using a camera positioned on a tripod outside of the test arena.

### Food motivation

To ensure that all animals were equally food motivated, we assessed the animals’ food motivation prior to starting the test. Each animal was led into the test arena separately. In three trials, the subjects were offered a handful of their usual mineral pellet food from a purple plastic bowl (19 cm diameter). To draw the subject’s attention towards the food, the bowl was shaken once before it was placed on the ground in front of the hurdles and subjects were released from the starting point. The animals were allowed to consume the food and were subsequently brought back to the starting point to initiate the next trial. Only animals which reliably approached and fed from the bowl in all three trials were included in the testing phase. Eleven llamas had to be excluded because they did not show sufficient feeding motivation and three llamas were excluded due to distress in the test arena; thus, 30 llamas proceeded to testing.

### Training of demonstrator animals

One to two animals from each farm were trained as demonstrator animals after they had participated in the study (all assigned to the control group). Demonstrator animals were selected based on the evaluation of the owner according to two criteria: (1) an intermediate position within the dominance hierarchy, and (2) generally food-motivated and easy to train. Only demonstrators of the same sex as the test subject were used. Since we did not conduct behavioural observations prior to the study, we relied on the owner’s assessment of dominance within the group and selected individuals of intermediate rank as these animals might be more accurately identified by the owners and were often the individuals that were described to be the most attentive and easily trainable. The demonstrators were trained individually using a stepwise training procedure. The llama was brought to the starting point (see Fig. 1) and the training started as soon as she/he started to pay attention to the presented food bowl. The baited food bowl was presented multiple times, starting in front of the metal hurdles, at slowly increasing distances to the starting position on the side of the hurdles before it was placed in the final reward location in the middle of the hurdles (see Fig. 1). Only if the demonstrator animal walked directly towards the food bowl (i.e. without any detours) in its final location in at least two repetitions within 60 s, they were used as conspecific demonstrators. We trained a total of six llamas, however, only four individuals reached this criterion and were subsequently used as demonstrators (2 M/2F; age: 3–18 yrs.; farm A: 2, farm B: 1, farm C: 1). All demonstrators reliably used the same route during the demonstration phase as they were trained to take (100% of demonstrations). The demonstrators needed 11.60 ± 2.88 s (range: 8–18 s) to reach the food bowl. Training of the animals and test sessions in which they were used as demonstrators took always place on the same day.

### Test procedure

Subjects that reached the food motivation criterion were pseudo-randomly assigned to one of the experimental groups (i.e. balanced within and across farms: farm A: 6 control, 3 conspecific, 2 human; farm B: 1 control, 3 conspecific, 5 human; farm C: 3 control, 4 conspecific, 3 human). Three test trials were conducted, which followed the same general procedure but differed in the demonstration prior to the first test trial (see below for description of groups). The two last test trials were always conducted without demonstrations. In the beginning of each trial, the handler and the animal stood at the starting point, while the experimenter stood behind the hurdles holding the food bowl in her hands. The bowl was then visibly baited by dropping a handful of pellets into the bowl, shaking the bowl once before placing it on the ground in the predefined position (see Fig. 1). The handler released the animal by letting go of the short lead rope at the moment that the experimenter placed the food bowl on the ground. The experimenter withdrew from the food bowl in a backward motion and remained passively (i.e. motionless and looking to ground) at the end of the arena in a central position. The handler likewise walked backwards from the starting position and remained passively during the trial. A trial ended either when the animal had reached the food bowl or after 60 s had passed. The handler then walked towards the animal, took the rope and led the animal back to the starting position.

### Control group (no demonstrator)

In the control group, the animals received no demonstration prior to the first test trial. The control group consisted of 10 llamas.

### Human demonstrator group

In the human demonstration group, the animals could observe the experimenter solve the detour prior to the first test trial. The experimenter baited the bowl (without shaking it), but then walked around the hurdles in a predefined way (randomised routes either to left or right side) towards the animal. The experimenter shortly stopped while standing in front of the animal (with a 1.5 m distance) looked at the animal’s head and then turned around and walked back towards the food bowl using the same route as before. Upon arriving at the food bowl, she shook the bowl once and placed it on the ground before the animal was released. The human demonstrator group consisted of 10 llamas (demonstration to right side: N = 5; left side: N = 5).

### Conspecific demonstrator group

In the conspecific demonstrator group, the test subject was led to the starting point together with a conspecific demonstrator. The experimenter baited the bowl (as described above) and the demonstrator animal was released while the test subject remained next to the handler. After the demonstrator animal reached the food bowl and finished eating the food reward, a second handler collected the demonstrator and led him/her to a central point in the middle of the arena next to the experimenter. The experimenter then went again to the food bowl, re-baited and shook it before placing it on the ground before the test subject was released. Following this first trial, the demonstrator was removed from the arena and the next trial started. The conspecific demonstrator group consisted of 10 llamas (demonstration to right side: N = 6, left side: N = 4).

### Ethical note

We adhered to the Guidelines for the Treatment of Animals in Behavioural Research and Teaching (Animal Behaviour 2020). The study was approved by the Animal Welfare Officer of the University of Giessen (approval number: JLU_kTV_3_2022). All owners were informed about the test procedure prior to starting the test. In case of an animal showing signs of stress (i.e. running and trying to escape from the test arena), the test was immediately terminated.

### Data scoring

All trials were videotaped (Sony HCR-CX190E Camcorder) and afterwards coded with the software BORIS (Friard and Gamba 2016). All analyses were conducted with R Version 4.0.2. (R Core Team 2014) using the packages ‘lme4’ (Bates and Maechler 2015), ‘survival’ (Therneau 2020), ‘survminer’ (Kassambara et al. 2020), ‘car’ (Fox and Weisberg 2019), ‘glmmTMB’ (Brooks et al. 2017).

We coded the following variables: success (yes, no), route (left, right), latency to feed (max. 60 s), duration of distraction, and duration of food-directed behaviours. We coded distraction as standing still but moving the head to either side to scan the environment. Food-directed behaviours were coded when an animal reached across the hurdles with its neck towards the food bowl. We needed to exclude two trials from the analyses, in which individuals managed to reach the food bowl by poking their head through the hurdle. To assess inter-observer reliability, 20% of the videos were coded by a second coder (Intra-class correlation coefficient (two-way, consistency: all > 0.88)).

### Analyses

To find out whether llamas improved their success after having observed demonstrations, we ran a generalised linear mixed model (GLMM) with a binomial error distribution and logit-link function (package: lme4). As response variable we entered the number of successful trials and the number of unsuccessful trials by using a response matrix with the *cbind*-function. Condition (factor: control, conspecific, human), age (z-transformed to a mean of 0 and a standard deviation of 1) and sex (factor: female, male) were included as predictors. To account for between-site variation, we included individual ID nested within farm ID as a random effect. Since the animals were only tested in one condition, no random slopes were identifiable. The model was strongly underdispersed (dispersion parameter = 0.186), which resulted in extremely wide confidence intervals (see Table S1). To overcome this issue, we fitted another GLMM with a poisson error distribution and log-link function. The number of successful trials was included as response variable (integer: 1–3) and the total number of trials as an offset term (log-transformed). We used the same predictors and random effect as in the original model. The poisson GLMM was not overdispersed (dispersion parameter = 0.942) and not affected by zero-inflation. The random effect exhibited a symmetrically distribution. We assessed model stability by dropping individuals one at a time from the data set, while fitting models based on these reduced datasets. The estimates were then compared to those of the model based on the full dataset, and the model proved to be of good stability (see Table S2). Collinearity was assessed using Variance Inflation Factors (VIFs) with the *vif*-function (package: car). We detected high collinearity between the two variables sex and age with VIFs of 1.59 and 1.53 respectively, and consequently, decided to remove the predictor sex from the model. This reduced model no longer had collinearity issues (all VIFs < 1.12). To avoid cryptic multiple testing (Schielzeth & Forstmeier 2009), we compared the full model (including condition and age as predictors) with a conceptual null model (including only age as predictor but with the same random effects structure as the full model) using a likelihood ratio test (LRT; Dobson & Barnett 2018). The significance tests that we report are all based on likelihood ratio tests (*drop1* function). Confidence intervals were obtained by means of parametric bootstrap (N = 1000 bootstraps) using the function *bootMer* of the lme4 package. The dataset for this analysis consisted of 30 observations from 30 llamas coming from three different farms.

Furthermore, we analysed whether llamas were quicker in solving the detour after having observed a demonstration and whether they improved their performance across trials. Accordingly, we ran a cox proportional hazards model (CPHM; package: survminer) using the latency to success as the response variable and an interaction between condition (factor: control, conspecific, human) and trial number (integer: 1–3) as predictors. Individual ID nested within farm ID was set as random effect and trial number was included as random slope. As an overall test for the effects of the predictors, we compared the full model with an intercept-only model using LRT. Model assumptions were assessed in Schoenfeld residual plots (*cox.zph* function) and revealed that the proportional hazards assumption was not violated (see Table S3). The dataset contained 88 observations from 30 llamas.

And finally, to find out whether certain behaviours during the test were associated with individual success, we ran two separate GLMMs with a beta error distribution and logit-link function (package: glmmTMB). The proportion of behaviours (GLMM1—time exhibiting distraction/total trial duration, and GLMM2—time showing food-directed behaviours/total trial duration) were used as response variables and age (z-transformed), success (binary: 0, 1), condition (factor: control, conspecific, human), and trial number (integer: 1, 2, 3) were set as predictors. To assess whether these behaviours were related to success in either condition, we included an interaction term between success and condition into the model. Individual nested within farm ID was used as random effect and trial number as random slope. The correlation between random slope and intercept needed to be excluded due to convergence issues. Again, we compared the full model with a null model (including only the intercept) using LRT. Using the *simulate*-function within the glmmTMB package, we obtained confidence intervals (N = 1000 bootstraps). Model stability was assessed as before (see Table S4 & S5), overdispersion (GLMM1: dispersion parameter = 0.94; GLMM2: dispersion parameter = 1.09) and collinearity (based on models lacking the interaction term; all VIFs < 1.15) was not an issue in either of the two models. The dataset contained 88 observations from 30 llamas.

## Results

### Success per condition

Fifteen llamas successfully solved the detour (48.4% of all llamas; control: 2/10; human: 7/10; conspecific: 6/10). During the conditions with demonstrations, nine out of 13 llamas that successfully found the food bowl, consistently used the same route as presented during the demonstration (human: 5 of 7; conspecific: 4 of 6); however, this finding was not different from chance level (two-sided binomial test: N = 13, p = 0.267). The side on which the majority of bystanders were standing during the trial did not affect the side, which was chosen to detour the hurdles (see Supplementary Material).

**Fig. 2:**
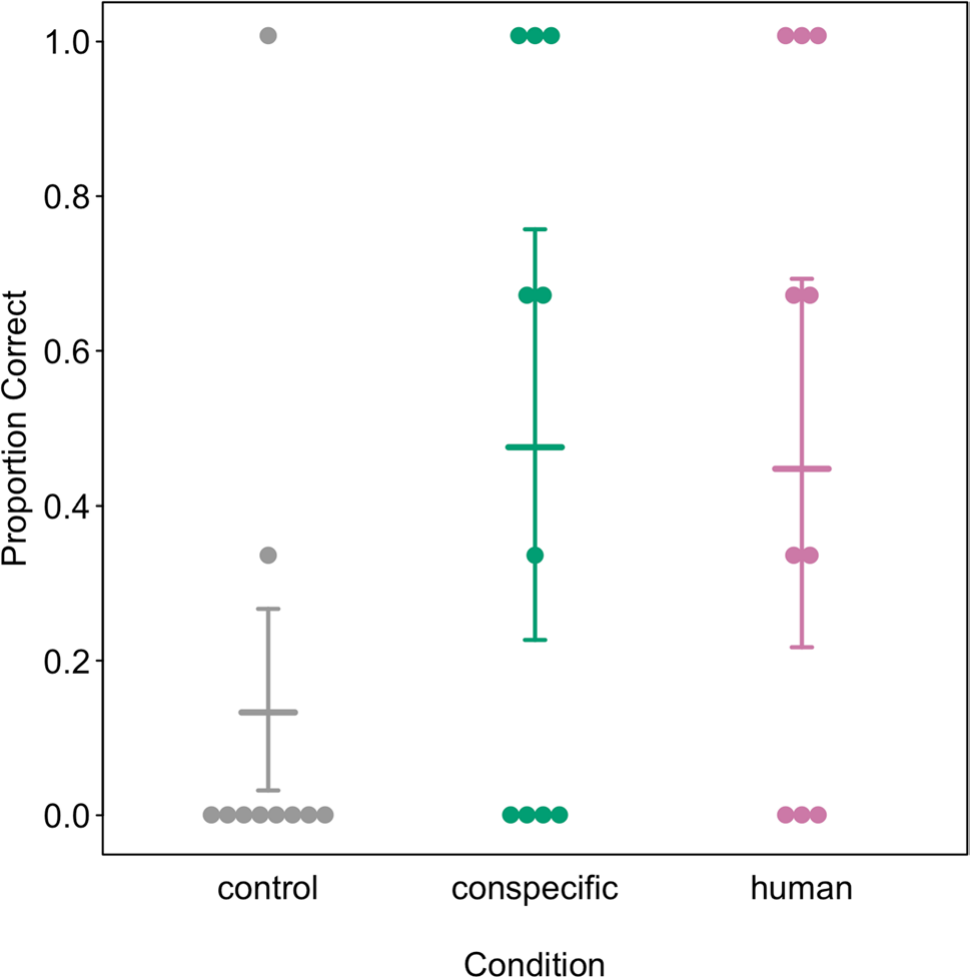
Proportion of successful trials per test condition. The coloured lines show the model estimates and the whiskers the confidence intervals. Points represent individual data

The full-null model comparison revealed that condition had a clear effect on the number of successful trials (see Table 1 and Fig. 2). Llamas were more successful after having observed a demonstration (conspecific: 1.4 ± 1.4, human: 1.3 ± 1.2 trials out of 3) compared to the control condition (0.4 ± 0.9 trials). Age had no observable effect on the performance in the test (see Table 1). The number of bystanders present during each trial as well as the side on which they were standing in relation to the hurdles did not differ between conditions or across trials (see Supplementary Material).

**Table 1:** Effects of condition and age on number of successful trials

Term	Estimate	SE	Lower CI	Upper CI	Chisq	df	P value
Intercept	− 2.021	0.510	− 3.457	− 1.262			^1^
Condition (conspecific)	1.274	0.577	0.265	2.868			
Condition (human)^2^	1.213	0.593	0.072	2.791	6.581	2	0.037
Age^3^	0.068	0.202	− 0.316	0.476	0.113	1	0.737

^1^not shown due to limited interpretabilitym

^2^indicated test refers to the overall effect of condition

^3^age was z-transformed to a mean of 0 and a standard deviation of 1. Original variable: 2–18 yrs

### Latency to success

**Fig. 3:**
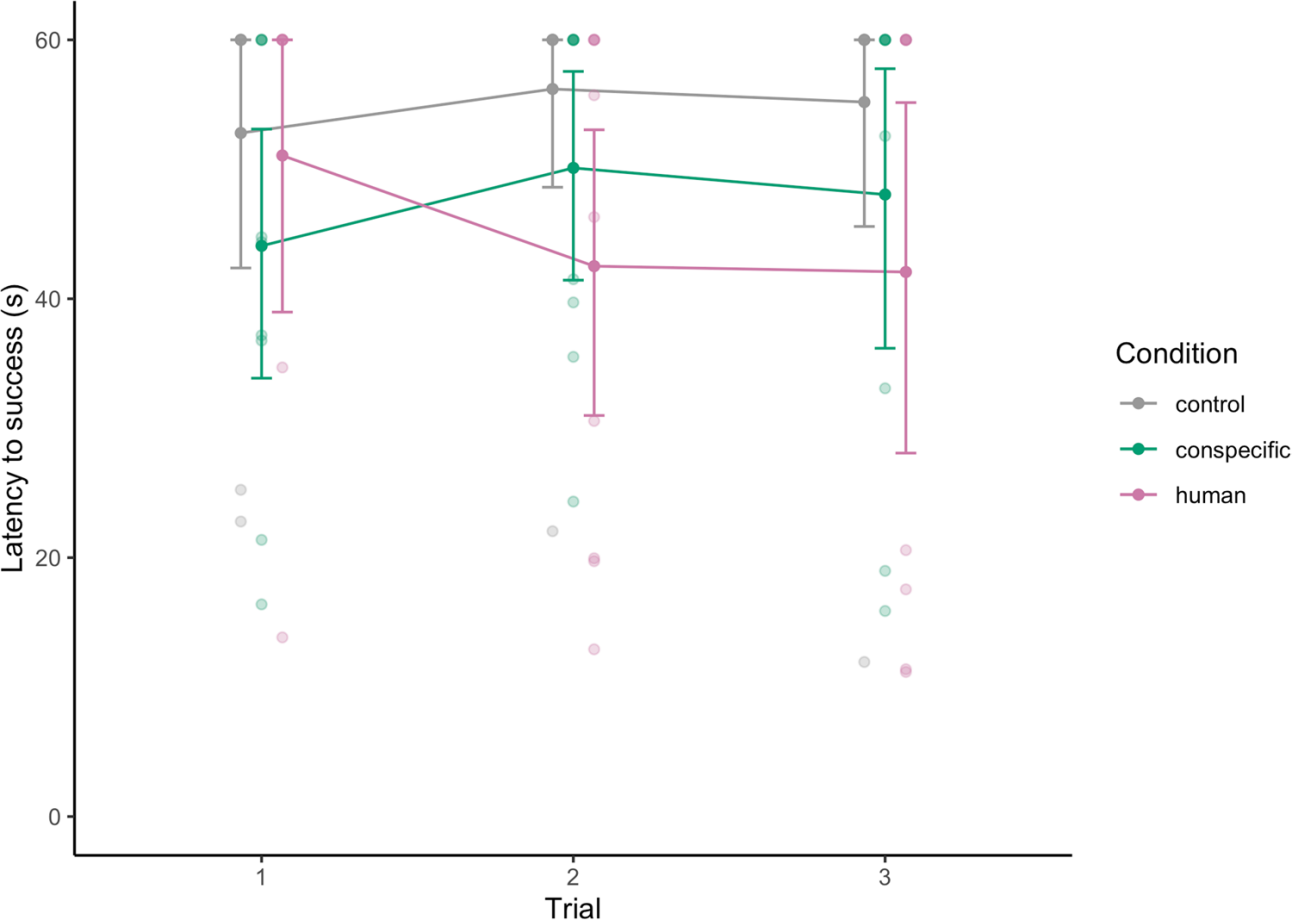
Latency to reach the food bowl across trials, plotted separately for each test group. Transparent points indicate raw individual data. Bold points and lines depict mean per group and error bars indicate the confidence limits for the mean. Note that for two individuals the data from the first trial was excluded because they reached the food bowl by inserting their head into the hurdles

The full-null model comparison revealed that the predictors had a clear effect on the latency to success (LRT: Chisq = 11.378, df = 5, p = 0.044). Accordingly, the interaction between condition and trial number revealed a tendency (see Table 2). In the human condition, the latency to success decreased across trials (trial 1: 51.07 ± 17.46 s; trial 2: 42.52 ± 19.60 s, trial 3: 42.07 ± 12.31 s) compared to the control condition (trial 1: 52.80 ± 15.18 s; trial 2: 56.20 ± 12.00 s; trial 3: 55.19 ± 15.20 s), while the latency to reach the food remained rather stable in the conspecific condition (trial 1: 44.08 ± 16.32 s; trial 2: 50.11 ± 13.53 s; trial 3: 48.05 ± 18.20; see Table 2 and Fig. 3).

**Table 2:** Results of CPHM with effects of trial number and condition on latency to reach the food reward

Term	Estimate	SE	Lower CI	Upper CI	Chisq	df	p-value
Trial	− 0.837	1.007	− 2.811	1.136			^1^
Condition (conspecific)	1.732	1.730	− 1.660	5.122			^1^
Condition (human)	− 0.560	1.850	− 4.186	3.065			^1^
Trial × condition (conspecific)	0.113	1.164	− 2.168	2.395			
Trial × condition (human)^2^	1.651	1.165	− 0.631	3.935	5.093	2	0.078

^1^not shown due to limited interpretability

^2^indicated test refers to the overall effect of condition

### Individual differences in success

**Fig. 4:**
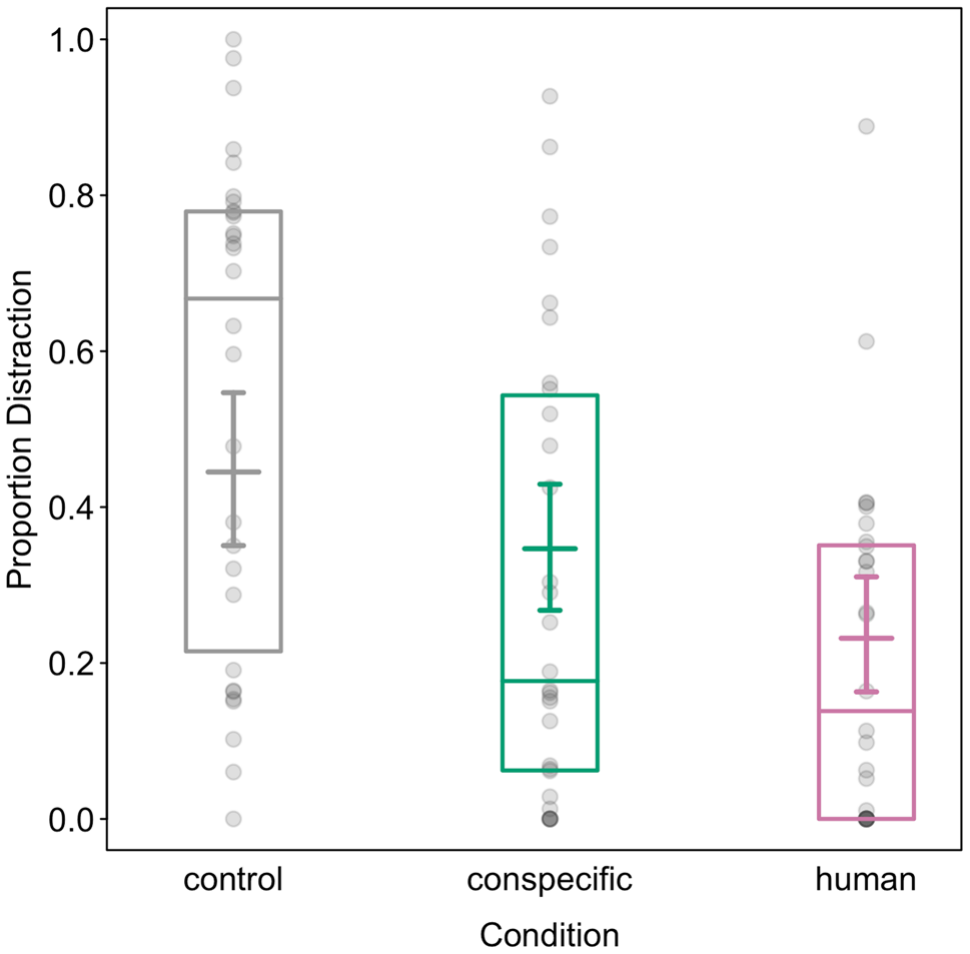
Proportion of distraction behaviours per test group. Points indicate raw data, coloured bars show medians, and boxes depict interquartile range. Coloured lines indicated model estimates and whiskers show confidence intervals

We found that distraction behaviours were clearly affected by the predictors (full-null comparison LRT: Chisq = 42.951, df = 7, p < 0.001). The interaction between success and condition, however, had no observable effect on the proportion of distraction behaviours (LRT: Chisq = 1.271, df = 2, p = 0.530). Consequently, we fitted a reduced model to assess the effects of the predictors as main effects (see Table S4 for results of full model). We found that distraction negatively affected success and older individuals exhibited more distraction behaviours than younger individuals (see Table 3). Furthermore, we found that distraction behaviours did not differ across trials but individuals were less distracted in the two test groups (conspecific: 0.305 ± 0.293 distraction/test duration; human: 0.207 ± 0.224) compared to the control group (0.541 ± 0.313; see also Fig. 4).

**Table 3:** Effects of success, condition, age, and trial number on the proportion of distraction behaviours in the test

Term	Estimate	SE	Lower CI	Upper CI	Chisq	df	p value
Intercept	0.391	0.331	− 0.252	1.042			^1^
Success	− 1.386	0.249	− 1.908	− 0.929	24.122	1	< 0.001
Condition (conspecific)	− 0.413	0.272	− 0.951	0.108			
Condition (human)^2^	− 0.978	0.286	− 1.566	− 0.405	10.551	2	0.005
Age^3^	0.361	0.118	0.132	0.609	8.827	1	0.003
Trial	− 0.109	0.131	0.871	1.463	0.691	1	0.406

^1^not shown due to limited interpretability

^2^indicated test refers to the overall effect of condition

^3^age was z-transformed to a mean of 0 and a standard deviation of 1. Original variable: 2–18 yrs

Food-directed behaviours were likewise strongly influenced by the predictors (full-null: LRT: Chisq = 22.625, df = 7, p = 0.002). Younger individuals showed more food-directed behaviours than older individuals while trial number had no observable effect on the proportion of food-directed behaviours (see Table 4). The interaction between success and condition proved to be significant; accordingly, individuals were more successful in the control (success: 0.328 ± 0.154, failure: 0.080 ± 0.126) and conspecific condition (success: 0.325 ± 0.225, failure: 0.151 ± 0.211) if they emitted a high proportion of food-directed behaviours (see Fig. S1). In the human condition, food-directed behaviours seem to have little effect on success (success: 0.114 ± 0.131, failure: 0.137 ± 0.145).

**Table 4:** Effects of success, condition, age and trial number on proportion of food-directed behaviours

Term	Estimate	SE	Lower CI	Upper CI	Chisq	df	p value
Intercept	− 2.372	0.362	− 3.169	− 1.696			
Success	1.882	0.520	0.889	2.947			^1^
Condition (conspecific)	0.385	0.369	− 0.253	1.127			^1^
Condition (human)	0.608	0.388	− 0.167	1.411			^1^
Age^2^	− 0.362	0.124	− 0.611	− 0.106	7.016	1	0.008
Trial	0.065	0.120	− 0.169	0.296	0.297	1	0.586
Condition (conspecific) x Success	− 0.804	0.639	− 2.047	0.405			
Condition (human) x Success^3^	-2.154	0.665	− 3.540	− 0.907	9.800	2	0.007

^1^not shown due to limited interpretability

^2^age was z-transformed to a mean of 0 and a standard deviation of 1. Original variable: 2–18 yrs

^3^the overall effect of the interaction is reported

## Discussion

We found that llamas were able to solve a spatial detour task, were more successful and solved the detour faster after having observed either a conspecific or a human performing the detour compared to a control condition without any demonstrations. In particular, during the first trial after the demonstration, llamas were quicker to reach the food in the conspecific group. In the human condition, the latency to reach the food decreased only across subsequent trials. Furthermore, behaviours observed during the test indicate that animals that displayed more distraction behaviours, hence paid less attention to the task, were less successful while individuals that showed more food-directed behaviours were more successful in the task.

In line with our hypothesis, we found that llamas were more successful in solving the detour following demonstrations; thus, indicating that llamas extracted some kind of information from the demonstrations instead of relying on individual learning for solving the task. The majority of llamas chose the same side to detour the hurdles as the demonstrators (69% of animals), however, this effect did not reach significance. Recent studies on social learning showed that different species seem to rely on various learning mechanisms to solve the detour task. For example, goats (Nawroth et al. 2016a) used the same side as demonstrated whereas other species, such as dogs or tortoises, did not, but were still susceptible to the facilitating effect of demonstrations (dogs: e.g. Mersmann et al. 2010; Pongrácz et al. 2001, 2003; tortoises: Wilkinson et al. 2010). Local or *stimulus enhancement* might explain the llamas’ performance, as the action of the demonstrator drew the subjects’ attention directly to the food bowl or to the area behind the hurdles.

Interestingly, the llamas showed relatively little improvement across trials, except for the human condition, in which the latency to reach the food clearly decreased across trials. In the conspecific condition, the llamas exhibit a lower latency already in the first trial and retrained the short latency across trials. While, in the control condition, the latency to reach the food was close to the maximum trial duration. This might indicate that social learning from conspecifics is more intuitive for llamas as they are more attentive to the actions of conspecifics, while social learning based on human demonstrations might require more repetitions or experience. A direct comparison between the human condition and the conspecific condition, however, is difficult due to procedural differences between demonstrations (i.e. starting point of demonstrators, food-directed behaviours). Nonetheless, the slow improvement across trials in the human condition might be due to using an unfamiliar human as the demonstrator. Being approached by an unfamiliar human might have initially elicited avoidance behaviours and only in subsequent trials, in which the unfamiliar experimenter no longer approached, the observed information from the demonstration was correctly applied by the llamas. Consequently, llamas might have performed better, if we had used a familiar human as demonstrator. Llamas are able to discriminate between humans based on familiarity (Taylor and Davis 1996) and familiarity can affect social learning performance (e. g. Figueroa et al. 2013; Guillette et al. 2016; but see Galef and Whiskin 2008). Alternatively, it is possible that llamas learned to ignore humans during specific activities (e.g. cleaning of barn) and only selectively attend to humans depending on the situation. Consequently, the llamas in our study might have assessed the situation and usefulness of attending to the actions of the human demonstrator first before exploiting the social information in subsequent trials.

Individual differences in success consistently emerge in studies assessing social learning abilities. Some individuals fail independent of whether they had the opportunity to observe a demonstration or not. Past studies have treated these individuals in different ways (i.e. exclude unsuccessful individuals: Pongrácz et al. 2001; Nawroth et al. 2016a; exclude individuals that solve task too quickly: Pongrácz et al. 2001; exclude unsuccessful trials: Mersmann et al. 2010) that could potentially influence outcomes. Biased input mechanisms (i.e. perceptual, attentive or motivational biases) can explain why some individuals learn better by observation than others (Heyes 2012). In particular, food motivation, attention, and neophobia, might account for inter-individual variation in the detour task. And indeed, we found that llamas that were more distracted and less focused on the food were also less successful. Only attentive individuals can extract information from demonstrations (Range and Huber 2007) but also persistence and goal-orientation can facilitate success (Pongrácz et al. 2021). Interestingly, distraction behaviours and food-related behaviours differed between test groups. Llamas were less distracted in the two demonstration groups compared to the control group, which might indicate that the demonstrations generally facilitated attention on the task. Furthermore, llamas were more successful, if they reached across the hurdles in the control and conspecific group, while no effect of reaching was found in the success rate in the human group. Whether this result is due to the fact that the animals were more focused on the route rather than the goal after the human demonstration is difficult to discern. In addition, age clearly affected distraction behaviours and food-motivation in the test. Older llamas were more distracted and less food-motivated than younger llamas; however, we found no general effect of age on success in the detour task. Furthermore, due to the separation distress that llamas exhibited when tested without visual access to conspecifics, we had to adjust our setup and place non-involved bystanders in an adjacent arena at the back of the test arena. While we found no effects of the number of bystanders or the side, on which they were standing, on the behaviour of subjects, it is still possible that they affected the subjects’ behaviour in subtler ways. Separation distress poses a major confounding factor in cognition studies that aim at testing highly gregarious species in an individual setup, as stress clearly affects cognitive abilities (Mendl 1999). Future studies with New World camelids therefore need to consider this issue and devise either careful training methods to habituate the animals to social separation during testing or test animals in a social setup while minimising potential confounding factors (e.g. by using visual barriers that prevent direct visual contact).

Considering that llamas were able to extract information based on the human demonstration, it might be argued that selection pressures during the domestication process have affected their sensitivity towards human behaviour. Llamas have been selectively bred to work in close contact with humans as pack animals (Mengoni Goñalons 2008; Wheeler 1995). Other animals that have been domesticated to live in close contact to humans and interact with them on a regular basis, such as dogs (e.g. Pongrácz et al. 2001), goats (Nawroth et al. 2016a), and geese (Fritz et al. 2000), are likewise able to extract information from observations of humans. Accordingly, it has been hypothesised that certain cognitive abilities (i.e. understanding of human social cues) might have been affected by domestication (e.g. Hare and Tomasello 2005; Udell et al 2010). Interestingly, however, cows (Stenfelt et al. 2022) and horses (Burla et al. 2018), failed to use information from human demonstrations in detour tasks. Whether this proficiency in extracting information from heterospecific demonstrations is indeed a result of selection for a cooperative and docile behaviour or rather caused by intense learning opportunities during every-day interactions cannot be answered with the current study. Nonetheless, our results raise several ideas for future research avenues. For example, testing equally socialised alpacas (*Vicugna pacos*), which are closely related to the llama but have been primarily selected for a fine fibre structure (Mengoni Goñalons and Yacobaccio 2006), might reveal whether domestication per se or specific selection for cooperative behaviours affected this sensitivity/attentiveness to human social cues. Ultimately, only by broadening the research approach to include other domesticated species, we will be able to understand how we have altered the behaviour of these animals during the process of domestication.

## Conclusion

We showed that llamas use information from humans and conspecifics in a spatial problem-solving task. Whether this social transmission of information was indeed based on emulation or rather local enhancement has to be investigated by future research. Taken together, these findings add support to the hypothesis that domestication for cooperativeness and close contact to humans is mirrored in an enhanced sensitivity towards human social cues. Individual differences in attention and food motivation need to be additionally considered when assessing social learning capacities. New World camelids prove to be promising model species for assessing the effects of domestication on cognitive abilities.

## Supplementary Information

Below is the link to the electronic supplementary material.

Supplementary file1 (PDF 89 KB)

Supplementary file2 (XLSX 20 KB)

Supplementary file3 (MP4 163839 KB)

## Data Availability

All data generated or analysed during this study are included in the supplementary information.
