# Supplementary material for: Llamas use social information from conspecifics and humans to solve a spatial detour task
Source: Anim Cogn. 2023 Jul 6;26(5):1623–33. doi: 10.1007/s10071-023-01808-8 (PMC10442258; doi:10.1007/s10071-023-01808-8)
Supplement: Supplementary file 1 — Supplementary file1 (PDF 89 KB) [file 10071_2023_1808_MOESM1_ESM.pdf]

Supplementary Material

Llamas use social information from conspecifics and humans to solve a spatial detour task

Annkatrin Pahl[a,b], Uta König von Borstel[c], Désirée Brucks[c]

[a]Department of Anthropology/Sociobiology, University of Göttingen, Göttingen, Germany
[b]Institute of Behavioural Physiology, Research Institute for Farm Animal Biology (FBN), Dummerstorf, Germany
[c]Animal Husbandry, Behaviour and Welfare Unit, Institute for Animal Breeding and Genetics, University of Giessen, Giessen, Germany

Table S1. Results from binomial GLMM with success and failure as response variable (using *cbind*-function) with minimum and maximum of estimates after dropping one individual at a time.

Term	Estimate	SE	Lower CI	Upper CI	Chisq	df	p-value	Min.	Max.
Intercept	-4.443	1.764	-23.579	-1.789			[1]	-5.446	-4.136
Condition (conspecific)	4.068	2.096	0.463	22.906				3.348	5.022
Condition (human)[2]	4.235	2.150	0.654	22.992	6.658	2	0.036	3.486	5.209
Age[3]	0.198	0.780	-1.640	2.345	0.064	1	0.800	2.453	3.653

[1] not shown due to limited interpretability
[2] test for overall effect of condition is depicted
[3] age was z-transformed to a mean of 0 and a standard deviation of 1. Original variable: 2-18 yrs.

Table S2. Model stability estimates of poisson GLMM with number of successful trials as response variable. Minimum and maximum estimates after dropping one individual at a time.

Term	Estimate	Min	Max
Intercept	-2.021	-3.397	-1.017
Condition (conspecific)	1.274	0.319	2.376
Condition (human)	1.213	-0.256	2.361
Age	0.068	0.000	0.394

Table S3. Test for proportional hazards assumption of the Cox regression model for the latency to success in llamas.

Term	Chisq	df	p-value
Trial	1.397	1	0.24
Condition	1.496	2	0.47
Trial x Condition	0.913	2	0.63
Global	3.123	5	0.68

Table S4. Results of full model for distracted behaviours (GLMM with beta error distribution) with minimum and maximum model estimates after dropping one individual at a time.

Term	Estimate	SE	Chisq	df	p-value	Min	Max
Intercept	0.476	0.346			[1]	0.184	0.685
Success	-1.937	0.572			[1]	-2.146	-1.430
Condition (conspecific)	-0.559	0.313			[1]	-0.772	-0.411
Condition (human)	-1.109	0.350			[1]	-1.326	-0.937
Age[2]	0.390	0.121	9.913	1	0.002	0.288	0.497
Trial	-0.119	0.133	0.797	1	0.372	-0.168	-0.031
Condition (consp) x Success	0.728	0.676				0.236	0.968
Condition (human) x Success[3]	0.622	0.687	1.271	2	0.530	0.121	1.091

[1] not shown due to limited interpretability

[2] age was z-transformed to a mean of 0 and a standard deviation of 1. Original variable: 2-18 yrs.

[3] test for overall effect of condition is depicted

Table S5. Model stability estimates of beta GLMM with proportion of food-directed behaviours as response variable. Minimum and maximum estimates after dropping one individual at a time.

Term	Estimate	Min	Max
Intercept	-2.372	-2.434	-2.207
Success	1.882	0.714	2.355
Condition (conspecific)	0.385	-0.007	0.596
Condition (human)	0.608	0.346	0.823
Age	-0.362	-0.413	-0.281
Trial	0.065	0.013	0.110
Condition (consp) x Success	-0.804	-1.179	0.462
Condition (human) x Success	-2.154	-2.594	-0.953

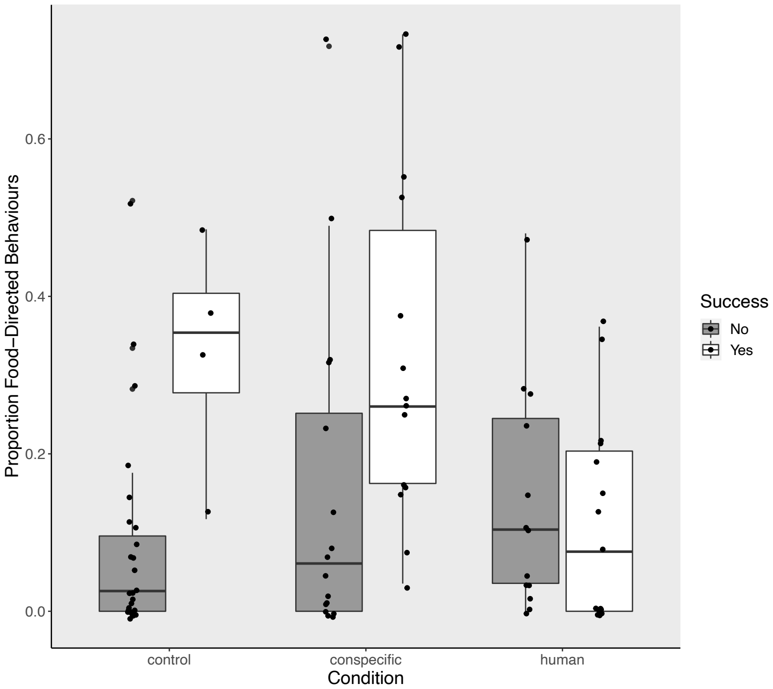

Figure S1. Proportion of food-directed behaviours (duration behaviour/ total trial duration) shown across test conditions. Success and failure within a trial are plotted separately (grey = failure; white = success). Black points represent raw data. Black bar depicts median, boxes indicate interquartile range and whiskers show upper and lower hinges.

Behaviour of bystanders

The number of bystanders present within 10m to the test arena during each trial was noted (mean ± SE: 1.84 ± 0.13 bystanders; range: 0-4). To find out whether the number of bystanders differed between conditions and trials, we ran a GLMM with a poisson error distribution; setting the number of bystanders (integer) as response variable and an interaction between condition (factor: control, conspecific, human) and trial (integer) as predictors. Individual ID was included as random effect and trial number as random slope. The model was underdispersed (dispersion parameter: 0.33) and of moderate stability (see Table S6).

A comparison between the full and null model (intercept-only model) revealed that the predictors had no observable effect (LRT: Chisq = 7.063, df = 5, p = 0.216); consequently, the number of bystanders did not differ between conditions or trials

Table S6. Model stability estimates of poisson GLMM with number of bystanders as response variable. Minimum and maximum estimates after dropping one individual at a time.

Term	Estimate	Min	Max
Intercept	0.211	-0.010	0.360
Condition (conspecific)	0.620	0.398	0.867
Condition (human)	0.609	0.467	0.853
Trial	-0.059	-0.116	0.036
Condition (consp) x Trial	-0.057	-0.145	0.044
Condition (human) x Trial	0.047	-0.042	0.101

Furthermore, we coded the duration of bystanders standing either to the left, middle or right in relation to the test arena. The bystander enclosure was divided into three equal areas and the duration of remaining in each area (i.e. majority of body within area) was coded separately for each bystander during each trial. Subsequently, we calculated the proportion of time remaining in each area in relation to the total trial duration and then used the mean across all bystanders to derive a group-level measure. We calculated a side ratio: (mean proportion time left – mean proportion time right) / (mean proportion time left + mean proportion time right). Consequently, a positive value indicated a left-side bias, a negative value a right-side bias and a value close to zero no side bias (mean ± SE: 0.18 ± 0.09).

To assess whether the bystanders' side bias differed across conditions or trials, we ran a GLMM with a beta error distribution and logit-link function (using the package glmmTMB (version 1.1.7; Brooks et al., 2017)) and set the absolute side ratio (i.e. transforming negative ratio to positive) as response variable. Prior to fitting the model, we transformed the side ratio to avoid having values of exactly zero or one in the data set (Smithson & Verkuilen, 2006). As predictors, we entered an interaction term between condition (factor) and trial number (integer), and included individual ID as random effect and trial number as random slope. The model was slightly overdispersed (dispersion parameter: 1.3) and of moderate stability (see Table S7).

The full-null (i.e. intercept-only) model revealed that the predictors had no clear effect on the side bias of bystanders (LRT: Chisq = 8.029, df = 5, p = 0.155); accordingly, the side biases did not differ between conditions or trials.

Table S7. Model stability estimates of beta GLMM with absolute side bias of bystanders as response variable. Minimum and maximum estimates after dropping one individual at a time.

Term	Estimate	Min	Max
Intercept	-0.204	-0.549	0.172
Condition (conspecific)	-0.885	-1.285	-0.542
Condition (human)	0.355	-0.018	0.700
Trial	0.316	0.191	0.461
Condition (consp) x Trial	0.490	0.347	0.631
Condition (human) x Trial	-0.105	-0.250	0.077

Finally, to analyse whether the side bias of the bystanders affected the subject's choice for a certain side to detour the hurdles, we calculated the concordance between the side bias of bystanders (i.e. > 0 = left, < 0 = right) and the selected side of the subject (i.e. 0 = different side selected, 1 = same side as bystanders, NA = subject not successful/ no bystanders present; mean ± SE: 0.43 ± 0.09). Subsequently, we ran a GLMM with a binomial error distribution using the glmmTMB package (and family: nbinom2 due to convergence issues with lme4). This concordance value (binary) was set as response variable and condition (factor) and trial number (integer) were used as predictors. Due to the low sample size (30 observations), the model was only of moderate stability (see Table S8).

The predictors had no observable influence on the concordance of choices between test subjects and bystanders (Full-null comparison: LRT: Chisq = 6.441, df = 3, p = 0.092).

Table S8. Model stability estimates of binomial GLMM with concordance of sides between subjects and bystanders as response variable. Minimum and maximum estimates after dropping one individual at a time.

Term	Estimate	Min	Max
Intercept	0.103	-0.173	0.458
Condition (conspecific)	-1.940	-19.48	-1.701
Condition (human)	-0.509	-0.643	-0.396
Trial	-0.060	-0.276	0.097

References

Brooks, M.E.; Kristensen, K; van Benthem, K.J.; Magnusson, A.; Berg, K.W.; Nielsen, A.; Skaug, H.J.; Maechler, M.; Bolker, B. (2017). glmmTMB Balances Speed and Flexibility Among Packages for Zero-inflated Generalized Linear Mixed Modeling. The R Journal, 9(2), 378-400. doi: 10.32614/RJ-2017-066.

Smithson, M.; Verkuilen, J. (2006). A better lemon squeezer? maximum-likelihood regression with beta-distributed dependent variables. Psychological Methods, 11:54–71.
